# The application value of quantitative analysis of orbital soft tissue parameters on plain CT scans in evaluating the activity of thyroid-associated ophthalmopathy

**DOI:** 10.3389/fendo.2026.1767565

**Published:** 2026-06-03

**Authors:** Gang Ji, Zheng-min Mo

**Affiliations:** Department of Radiology, Mian Yang Wanjiang Eye Hospital, Mianyang, Sichuan, China

**Keywords:** computed tomography, coronal scan, exophthalmos, extraocular muscles, intraorbital fat, lacrimal gland, thyroid-associated ophthalmopathy

## Abstract

**Purpose:**

Using plain orbital computed tomography (CT) scan images, we quantitatively extracted indicators such as diameters, cross-sectional areas, volumes, and densities of intraorbital soft tissues to investigate the feasibility of these metrics for assessing inflammatory activity status in patients with thyroid-associated ophthalmopathy (TAO).

**Methods:**

This study retrospectively recruited 31 patients with active TAO, 46 with inactive TAO, and 46 normal controls between April 2022 and October 2025 (note: counts of inactive TAO patients and normal controls overlapped due to contralateral eye classification in TAO patients). Clinical and imaging data were collected, and eyes were stratified into three groups: 50 active TAO eyes, 79 inactive TAO eyes, and 87 normal eyes. Orbital CT scans and 3D image analysis (3D Slicer 5.7.0) were performed to measure exophthalmos, axial length, globe width, cross-sectional areas (CSA) of individual/total extraocular muscles (EOMs), lacrimal gland CSA/volume (LGV), optic nerve CSA, intraorbital fat thickness/volume (IOFV), and densities of lacrimal gland, vitreous, and intraorbital fat. Ratios of LGV, IOFV, lacrimal gland CSA, and total EOMs CSA (OM) to total orbital volume (TV)/CSA (TOA) were calculated. The Kruskal–Wallis *H* test was used for intergroup comparisons, with pairwise Mann–Whitney *U* tests and Bonferroni correction (*α*=0.017) applied for significant parameters. Point-biserial correlation was used to quantify associations with active TAO, and receiver operating characteristic (ROC) curve analysis (maximum Youden index) was used to evaluate diagnostic value.

**Results:**

This study included 67 TAO patients (men:women = 35:32) with a 92.5% (62/67) bilateral involvement rate. Kruskal–Wallis *H* test showed significant differences among the active TAO, inactive TAO, and control groups in OM, CSA of the superior/inferior/medial rectus muscles, superior/inferior oblique muscles, TOA, lacrimal gland CSA, Intraorbital fat density, IOFV, vitreous density, lacrimal gland density, and the ratios of IOFV/TV, OM/TOA, and lacrimal gland CSA/TOA (all *p*<0.05). Mann–Whitney *U* tests revealed that patients with active TAO had significantly higher superior/medial rectus CSA, OM, Intraorbital fat density, and OM/TOA ratio than those in the inactive TAO and control groups (all *p* < 0.017). Point-biserial correlation analysis confirmed positive correlations between these five parameters and active TAO status (*r* > 0; all *p* < 0.05). ROC curve analysis demonstrated their diagnostic efficacy for active TAO: Intraorbital fat density ≥ − 78.5 HU (area under the curve [AUC] = 0.830, sensitivity: 76.0%, specificity: 89.2%), OM/TOA ratio ≥ 0.301 (AUC=0.829, sensitivity: 86.0%, specificity: 70.5%), superior rectus CSA ≥ 49.115 mm^2^ (AUC=0.812, sensitivity: 64.0%, specificity: 87.3%), OM ≥ 254.12 mm^2^ (AUC=0.825, sensitivity: 68.0%, specificity: 82.5%), and internal rectus CSA ≥ 43.525 mm^2^ (AUC=0.749, sensitivity: 58.0%, specificity: 84.3%) (all *p* < 0.001).

**Conclusions:**

The measurement of the CSA of the superior rectus muscle at 7 mm posterior to the orbital globe, the CSA of the medial rectus muscle, OM, Intraorbital fat density, and the OM/TOA ratio via plain CT scan serves as a reliable and feasible technical approach for evaluating disease activity in patients with TAO in primary ophthalmic healthcare institutions.

## Introduction

Thyroid-associated ophthalmopathy (TAO) is an organ-specific autoimmune disorder associated with thyroid dysfunction, primarily linked to autoimmune thyroid conditions such as Graves’ ophthalmopathy (GO) and Hashimoto’s thyroiditis (1). It is characterized by inflammatory proliferation and fibrosis of the extraocular muscles and orbital adipose tissue and exhibits a predilection for women, with a peak age of onset between 40 and 60 years (2, 3). In the USA, the age-adjusted incidence rate of TAO is approximately 16 cases per 100,000 person-years among women, compared with 2.9 cases per 100,000 person-years among men (4). The clinical spectrum of TAO is broad, encompassing ocular manifestations such as eyelid retraction, exophthalmos, conjunctival edema, periorbital edema, and ocular motility dysfunction, ranging from mild presentations to severe, vision-threatening conditions (5).

Orbital computed tomography (CT) findings in patients with TAO are predominantly characterized by proptosis and extraocular muscle involvement. Approximately 76% to 90% of TAO patients exhibit unilateral and/or bilateral extraocular muscle involvement, with the inferior rectus muscle being the most commonly affected, followed by the medial rectus, superior rectus, and lateral rectus muscles (6). Accurate assessment of clinical activity is critical for determining the optimal timing of intervention in patients with TAO. The Clinical Activity Score (CAS) is the most widely utilized metric for evaluating inflammatory activity; however, it relies on subjective symptoms and patient compliance (7), rendering it highly subjective and prone to confounding factors. Advances in imaging technologies have enabled the objective quantification of pathological lesions. CT, characterized by its high resolution and reproducibility, offers unique advantages in delineating changes in orbital soft tissues. Quantitative analysis of the volume and density of extraocular muscles (EOMs), lacrimal glands, and adipose tissue via three-dimensional (3D) reconstruction techniques not only reflects the extent of lesion involvement but also reveals the severity of the disease process. Integration of multiple quantitative parameters into statistical models can effectively differentiate between active and inactive disease phases, thereby enhancing diagnostic accuracy. Byun et al. (8) performed CT scans on patients with TAO to quantify the volume and density of orbital soft tissues. Their findings demonstrated that this approach constitutes a reliable and feasible technique for evaluating the inflammatory activity status in TAO patients. Consequently, CT-based quantitative analysis holds substantial clinical value in guiding therapeutic interventions and is particularly well suited for dynamic monitoring and disease assessment of TAO patients in primary healthcare settings.

In recent years, with the further advancement of China’s hierarchical medical system, the diagnosis and treatment of common diseases such as TAO have gradually been shifted to primary healthcare institutions (9). For primary healthcare institutions, medical equipment allocation is relatively limited. High-end imaging modalities such as magnetic resonance imaging (MRI) and positron emission tomography/computed tomography (PET/CT) have low penetration rates, coupled with high acquisition and operational costs, which hinder their widespread implementation. In contrast, CT systems are widely accessible in primary healthcare institutions, characterized by ease of operation, high examination efficiency, and favorable cost-effectiveness, thus conferring greater accessibility and clinical utility. Currently, there is a paucity of studies investigating the use of noncontrast CT for assessing TAO activity. Whether quantitative analysis of intraorbital soft tissue parameters via CT can serve as a quantitative criterion for evaluating TAO activity remains inconclusive and warrants further investigation. The present study aims to quantitatively measure various intraorbital soft tissue parameters using orbital CT images, explore their feasibility in assessing TAO inflammatory activity, and employ CAS as the clinical reference for stratifying the disease status of TAO patients.

## Materials and methods

### Study subjects

This study retrospectively included 31 active TAO patients, 46 inactive TAO patients, and 46 normal controls (April 2022–October 2025; note: counts of inactive TAO patients and normal controls had overlaps due to contralateral eye classification of TAO patients). Of the 31 active TAO patients, 12 had unilateral active involvement (2 with contralateral normal eyes, 10 with contralateral inactive eyes); 2 inactive TAO patients had contralateral normal eyes. Clinical and imaging data were collected, and eyes were stratified into three groups: 50 active TAO eyes, 79 inactive TAO eyes, and 87 normal eyes. This study was formally approved by the ethics committee of Mian Yang Wanjiang Eye Hospital, with IRB approval number 2025-Article (04). All image acquisition, processing, and analysis procedures involved in this study strictly adhere to the principles outlined in the Declaration of Helsinki. All patients underwent orbital CT scans prior to definitive diagnosis, with images acquired using standardized equipment and consistent scanning parameters. Correlation analyses were performed by measuring the following parameters: exophthalmos, axial length, ocular width, lacrimal gland cross-sectional area (CSA), the density and volume of the lacrimal gland and intraorbital fat, intraorbital fat thickness, vitreous density, CSA of each type of EOMs, as well as the ratios of intraorbital fat volume (IOFV) to total orbital volume (TV), total lacrimal gland volume (LGV) to TV, total CSA of lacrimal gland to total CSA of orbital (TOA), and total CSA of EOMs (OM) to TOA. These measurements were integrated with CAS for correlation analyses. All data were measured at a 7-mmeter posterior plane to the globe in the coronal orientation except exophthalmos, axial length and width, lacrimal gland, vitreous body, and inferior oblique muscle measurements.

Patients with TAO were diagnosed in accordance with the Bartley criteria (10). The following participants were excluded: those aged < 18 years, individuals with a prior history of ophthalmic medical or surgical interventions (including systemic steroid therapy, anti-inflammatory or immunomodulating agents [including teprotumumab], orbital radiotherapy, or surgical decompression), pregnant women, and subjects with incomplete CT imaging data. The normal control group comprised individuals who attended the clinic for mild periorbital soft tissue contusion or routine health screenings, with no self-reported history of systemic or ophthalmic diseases, except for mild-to-moderate myopia. TAO patients with a CAS ≥ 3 were categorized as the active TAO group, whereas those with CAS < 3 were assigned to the inactive TAO group. CAS criteria and scoring system include spontaneous retrobulbar pain, pain on eye movement, eyelid hyperemia, eyelid edema, conjunctival hyperemia, chemosis, and caruncular swelling, each assigned 1 point, yielding a maximum total score of 7 points. All enrolled participants underwent a comprehensive ophthalmic examination, and demographic and clinical data (including age, gender, CAS, and duration of disease) were extracted from electronic medical records. All enrolled subjects provided written informed consent prior to undergoing examinations.

### CT scanning and data measurement protocols

All enrolled participants underwent orbital CT using a 16-slice multidetector CT scanner (Philips MX 16-Slice; Philips Medical Systems Suzhou Co. Ltd., Suzhou, China) without intravenous contrast administration. Acquisition was performed using continuous spiral scanning, with the participant’s head aligned parallel to the Frankfurt horizontal plane. The scan was initiated at the level of the infraorbital margin and extended cephalad to the superior orbital margin, using the orbitomeatal line as the reference baseline. Scanning parameters were standardized as follows: tube current, 250 mA; tube voltage, 120 kV; collimation, 0.75 mm; pitch factor, 0.60; gantry rotation time, 1 s/rotation; reconstructed slice thickness, 1.5 mm; interslice gap, 0.75 mm; and image matrix, 768 × 768. Soft tissue window settings were set to a width of 500 HU and a level of 60 HU, while bone window settings were configured with a width of 3,000 HU and a level of 400 HU.

After completion of the routine spiral CT protocol described above, all acquired orbital images were immediately processed via high-resolution thin-slice reconstruction with the following parameters: reconstructed slice thickness, 0.75 mm; reconstruction increment, 0.375 mm; bone window matrix, 1,024 × 1,024; and soft tissue window (width: 500 HU, level: 60 HU) matrix, 512 × 512. Reconstructed images were then transmitted to the Picture Archiving and Communication System (PACS) workstation for centralized storage. Quantitative measurements of orbital soft tissue metrics—including linear dimensions, density, cross-sectional area, and volume—were performed using 3D Slicer software (version 5.7.0).

**Figure 1:**
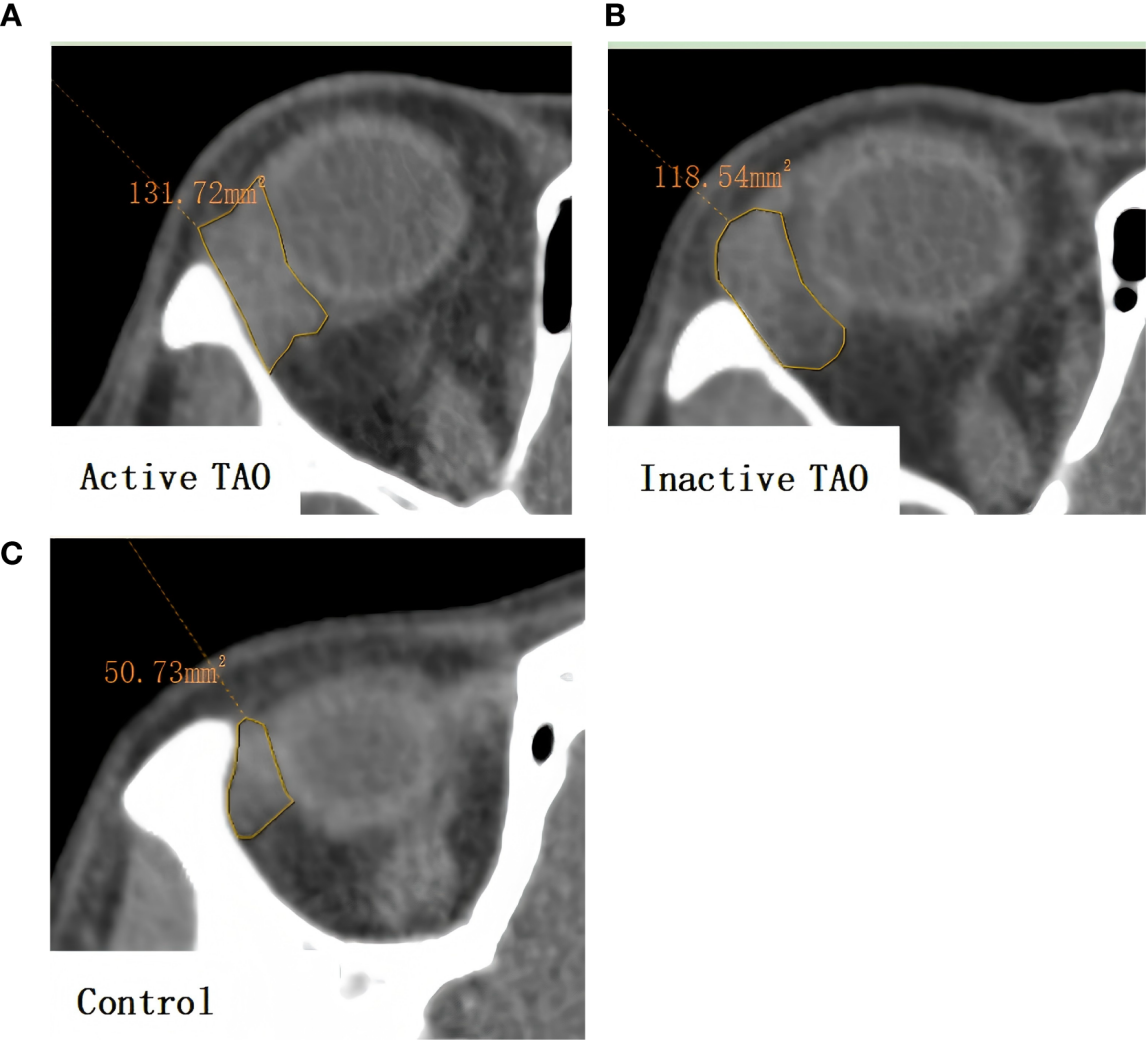
**(A–C)** Comparison chart of the cross-sectional area of the lacrimal glands among three groups of patients. The circled areas in the images, from left to right, indicate the CSA of the lacrimal glands in patients with active TAO, inactive TAO, and the control group, respectively. As observed from the figure, patients with active-phase TAO exhibit a significant increase in the CSA of the lacrimal gland. CSA, cross-sectional area; TAO, thyroid-associated orbitopathy.

Orbital boundaries were anatomically defined as follows: (1) anterior orbital boundary: in axial sections, a straight line connecting the most anterior points of the lateral and medial orbital rims; (2) superior and inferior orbital boundaries: in sagittal sections, demarcated by the bony orbital plates; discontinuous segments were bridged with straight lines to ensure a closed boundary; (3) posterior orbital boundary: in sagittal sections, corresponding to the anterior margin of the optic canal; (4) medial and lateral orbital boundaries: in axial sections, demarcated by the bony orbital plates; discontinuous segments were bridged with straight lines to ensure a closed boundary. The most anterior anatomical landmark of the medial orbital wall is the anterior lacrimal crest; that of the lateral orbital wall is the orbital surface of the zygomatic bone; that of the superior orbital wall is the orbital plate of the frontal bone; and that of the inferior orbital wall is the infraorbital foramen. Segmentation of CT images was performed using the Segment Editor module in 3D Slicer software, employing a threshold-based segmentation algorithm. Soft tissue window settings were applied to segment three key orbital components: lacrimal gland, orbital fat, and total orbital volume. At the continuous level, the edge of the lacrimal gland was manually traced to separate it from the extraocular muscles and tendon (**Figure 1**). Coronal orbital CT imaging was utilized to perform quantitative measurements of the lacrimal gland, including its maximum slice diameter, cross-sectional area, and mean density. On coronal reconstructed CT images, at 7mm posterior to the globe, the diameter and cross-sectional area of the thickest slices of the superior rectus, inferior rectus, medial rectus, lateral rectus, and superior oblique muscles were measured. On obliquely sagittal reconstructed CT images, at 7 mm posterior to the globe, the diameter and cross-sectional area of the thickest slice of the inferior oblique muscle were measured. The threshold ranges were defined as follows: lacrimal gland tissue: − 30 to + 100 HU; orbital adipose tissue: − 200 to − 30 HU; total orbital volume: − 200 to + 100 HU. Both conventional multislice computed tomography (MSCT) images and postprocessed thin-slice reconstructed images were quality-controlled to ensure clear visualization of the orbital soft tissues and bony orbital walls. Image analysis was conducted by two board-certified radiologists with ≥ 5 years of clinical experience using a double-blind protocol (neither radiologist was aware of the patient’s clinical grouping). In cases of interobserver discrepancy, consensus was reached through joint review to finalize measurements.

### Statistical analysis

IBM SPSS Statistics (version 27.0) was used for all analyses. Continuous demographic variables (age) were compared among the three groups via one-way analysis of variance (ANOVA), while categorical variables (gender) were analyzed using Pearson’s Chi-square test. Nonparametric variables were compared with the Kruskal–Wallis *H* test. All orbital metrics were standardized to unified units: linear dimensions (mm), CSA (mm^2^), volume (mm^3^), and density (HU). For Kruskal–Wallis *H* test results with statistical significance (*p* < 0.05), pairwise comparisons were performed using the Mann–Whitney *U* test, with Bonferroni correction applied (adjusted *α* = 0.017) to control for type I error. Spearman’s rank correlation coefficient was used to evaluate monotonic associations between TAO activity-related metrics and binary activity status (active vs. inactive), complemented by point-biserial correlation for binary–continuous associations. Receiver operating characteristic (ROC) curve analysis was employed to assess the diagnostic efficacy of each metric, with the optimal cut-off value determined by maximizing the Youden index (*J* = sensitivity + specificity − 1).

## Results

### Baseline clinical characteristics of the three study groups

In the TAO cohort, the male-to-female ratio was 35:32, with bilateral orbital involvement observed in 92.5% of patients (62/67). The mean CAS was 3.9 in the TAO active subgroup and 1.8 in the TAO inactive subgroup. No statistically significant differences were detected in gender distribution (Pearson’s Chi-square test; *p* > 0.05) or age distribution (one-way ANOVA; *p* > 0.05) across the three groups, indicating that the three patient groups were matched for key demographic or clinical variables (details are presented in **Table 1**). Disease duration was significantly longer in the inactive TAO group than in the active group (4.07 ± 6.59 vs. 1.12 ± 1.91; *p* < 0.001; **Table 1**).

**Table 1:** Basic clinical characteristics of the three groups of patients.

Characteristics	Active TAO	Inactive TAO	Controls	*P*-value
Number of patients (*N*)	31	46	46	–
Orbital (*N*)	50	79	87	–
Age (years)^a^	55.7 ± 10.8	54.3 ± 13.2	51.9 ± 11.6	0.638
Gender (men:women)	19:12	23:23	24:22	0.602
CAS	3.9 (3–6)	1.8 (0–2)	0	–
Duration of disease (years)	1.12 ± 1.91	4.07 ± 6.59	–	< 0.001

*TAO*, thyroid-associated orbitopathy; *CAS*, Clinical Activity Score.

^a^ Data are expressed as mean ± SD.

### Kruskal–Wallis H test

The results showed statistically significant differences among the three groups in terms of exophthalmos, the CSA of the superior and inferior rectus muscles, the CSA of the superior and inferior oblique muscles, OM, the CSA of the lacrimal gland, IOFV, intraorbital fat density, vitreous density, lacrimal gland density, IOFV/TV ratio, OM/TOA ratio, and the ratio of lacrimal gland CSA to TOA (*p* < 0.05; see **Tables 2**, **3** for details).

**Table 2:** Comparison of orbital soft tissue linear dimensions, area, volume, and density measurements among the three groups.

Parameters	Active TAO	Inactive TAO	Controls	*P*-value
Exophthalmos (mm)	20.50 (19.03–21.98)	20.45 ± 3.10	15.46 ± 1.80	< 0.001^*^
Axial length (mm)	23.35 (22.45–24.25)	23.29 ± 0.97	23.61 ± 0.83	0.188
Axial width (mm)	24.06 ± 1.61	24.19 ± 1.28	24.01 ± 1.04	0.750
CSA of the superior rectus muscle (mm^2^)	59.11 (36.92–81.30)	39.94 (31.14–48.75)	30.85 ± 7.45	< 0.001^*^
CSA of the inferior rectus muscle (mm^2^)	63.07 (37.27–88.86)	51.87 (32.74–70.99)	33.83 ± 8.17	< 0.001^*^
CSA of the internal rectus muscle (mm^2^)	50.33 (32.07–68.58)	35.63 (23.51–47.76)	27.75 (23.55–31.94)	< 0.001^*^
CSA of the lateral rectus muscle (mm^2^)	34.58 (26.76–42.39)	32.47 (25.88–39.07)	33.23 (28.21–38.23)	0.319
CSA of the superior oblique muscle (mm^2^)	19.99 ± 7.11	15.71 (12.35–19.07)	12.10 (9.35–14.85)	< 0.001^*^
CSA of the inferior oblique muscle (mm^2^)	48.02 (31.02–65.01)	41.50 (30.70–52.30)	27.17 (20.78–33.55)	< 0.001^*^
OM (mm^2^)	298.77 ± 83.31	237.23 (191.95–282.51)	169.18 ± 28.57	< 0.001^*^
CSA of the lacrimal gland (mm^2^)	91.20 ± 26.26	81.67 (64.31–99.03)	66.44 (53.36–79.51)	< 0.001^*^
CSA of the optic nerve (mm^2^)	23.31 ± 5.71	21.90 (18.25–25.55)	23.08 (20.30–25.86)	0.376
IOFV(mm^3^)	6920.85 (5304.56–8537.13)	8,966.41 ± 2,638.90	7640.08 (6790.41–8489.76)	< 0.001^*^
LGV(mm^3^)	351.28 (163.36–539.19)	261.85 (120.40–403.30)	298.76 (180.78–416.74)	0.173
Orbital adipose tissue thickness (mm)	4.20 (3.70–4.70)	4.75 ± 1.18	4.47 ± 1.00	0.072
Intraorbital fat density (HU)	-74.00 (-78.00–-70.00)	− 85.61 ± 8.48	-85.00 (-89.00–-81.00)	< 0.001^*^
Vitreous body density (HU)	11.50 (9.50–13.50)	11.00 (8.50–13.50)	13.41 ± 2.92	< 0.001^*^
Lacrimal Gland Density (HU)	30.56 ± 12.21	28.00 (19.00–37.00)	33.50 (24.85–42.15)	0.019^*^

*TAO*, thyroid-associated orbitopathy; *CSA*, cross-sectional area; *OM*, total cross-sectional area of extraocular muscles; *IOFV*, intraorbital fat volume; *LGV*, lacrimal gland volume; *HU*, Hounsfield unit. ^*^p < 0.05. Data are presented as mean ± SD (normal distribution) or median (IQR) (non-normal distribution).

**Table 3:** Comparative analysis of IOFV and LGV as ratios to TV among three groups versus the corresponding ratios of OM and lacrimal gland CSA to TOA.

Parameters	Active TAO	Inactive TAO	Control	*P*-value
IOFV/TV	0.30 (0.24–0.37)	0.39 ± 0.10	0.33 (0.30–0.37)	< 0.001^*^
LGV/TV	0.01 (0.01–0.02)	0.01 (0.00–0.02)	0.01 (0.01–0.02)	0.249
OM/TOA	0.38 ± 0.08	0.27 (0.21–0.33)	0.27 ± 0.04	< 0.001^*^
CSA of the lacrimal gland/TOA	0.12 ± 0.04	0.10 (0.08–0.12)	0.11 (0.08–0.13)	0.032^*^

*TAO*, thyroid-associated orbitopathy; *IOFV*, intraorbital fat volume; *LGV*, lacrimal gland volume; *TV*, total orbital volume; *OM*, total cross-sectional area of extraocular muscles; *TOA*, total orbital cross-sectional area; *CSA*, cross-sectional area. ^*^p < 0.05. Data are presented as mean ± SD (normal distribution) or median (IQR) (non-normal distribution).

### Rank sum test (Mann–Whitney U test)

Further pairwise comparisons of the indicators with significant differences in the above statistical analysis were conducted using the rank sum test (Mann–Whitney *U* test) among the three groups. The results showed that the CSA of the superior rectus and medial rectus muscles, OM, orbital fat density, and the ratio of EOMs CSA to TOA in the active TAO group were significantly higher than those in the inactive TAO group and the control group, with statistically significant differences (*p* < 0.017; see **Tables 4**, **5**), suggesting that these indicators are closely related to the inflammatory activity state of TAO. Additionally, exophthalmos, the CSA of the superior rectus, inferior rectus, superior oblique, and inferior oblique muscles, OM, the CSA of the lacrimal gland, and the vitreous density in both TAO groups were significantly higher than those in the control group (*p* < 0.017; see **Tables 5**, **6**), indicating that these indicators are closely associated with the occurrence of TAO.

**Table 4:** Mann–Whitney *U* test between the active TAO group and the inactive TAO group.

Parameters	Active TAO	Inactive TAO	*P*-value
Exophthalmos (mm)	20.50 (19.03–21.98)	20.45 ± 3.10	0.306
CSA of the superior rectus muscle (mm^2^)	59.11 (36.92–81.30)	39.94 (31.14–48.75)	< 0.001^*^
CSA of the inferior rectus muscle (mm^2^)	63.07 (37.27–88.86)	51.87 (32.74–70.99)	0.035
CSA of the internal rectus muscle (mm^2^)	50.33 (32.07–68.58)	35.63 (23.51–47.76)	0.014^*^
CSA of the superior oblique muscle (mm^2^)	19.99 ± 7.11	15.71 (12.35–19.07)	0.023
CSA of the inferior oblique muscle (mm^2^)	48.02 (31.02–65.01)	41.50 (30.70–52.30)	0.106
OM (mm^2^)	298.77 ± 83.31	237.23 (191.95–282.51)	< 0.001^*^
CSA of the lacrimal gland (mm^2^)	91.20 ± 26.26	81.67 (64.31–99.03)	0.046
IOFV(mm^3^)	6920.85 (5304.56–8537.13)	8,966.41 ± 2,638.90	0.002^*^
Intraorbital fat density (HU)	-74.00 (-78.00–-70.00)	− 85.61 ± 8.48	< 0.001^*^
Vitreous body density (HU)	11.50 (9.50–13.50)	11.00 (8.50–13.50)	0.784
Lacrimal gland density (HU)	30.56 ± 12.21	28.00 (19.00–37.00)	0.127
IOFV/TV	0.30 (0.24–0.37)	0.39 ± 0.10	< 0.001^*^
OM/TOA	0.38 ± 0.08	0.27 (0.21–0.33)	< 0.001^*^
CSA of the lacrimal gland/TOA	0.12±0.04	0.10 (0.08–0.12)	0.009^*^

*TAO*, thyroid-associated orbitopathy; *CSA*, cross-sectional area; *EOMs*, extraocular muscles; *IOFV*, intraorbital fat volume; *LGV*, lacrimal gland volume; *HU*, Hounsfield unit; *TV*, total orbital volume; *OM*, total cross-sectional area of extraocular muscles; *TOA*, total orbital cross-sectional area. ^*^p < 0.017 (the *p*-values were corrected using the Bonferroni method [corrected *α* = 0.05/3 ≈ 0.017; *p* < 0.017 was considered significant]). Data are presented as mean ± SD (normal distribution) or median (IQR) (non-normal distribution).

**Table 5:** Mann–Whitney *U* test between the TAO active group and the control group.

Parameters	Active TAO	Control	*P*-value
Exophthalmos (mm)	20.50 (19.03–21.98)	15.46 ± 1.80	< 0.001^*^
CSA of the superior rectus muscle (mm^2^)	59.11 (36.92–81.30)	30.85 ± 7.45	< 0.001^*^
CSA of the inferior rectus muscle (mm^2^)	63.07 (37.27–88.86)	33.83 ± 8.17	< 0.001^*^
CSA of internal rectus muscle (mm^2^)	50.33 (32.07–68.58)	27.75 (23.55–31.94)	< 0.001^*^
CSA of the superior oblique muscle (mm^2^)	19.99 ± 7.11	12.10 (9.35–14.85)	< 0.001^*^
CSA of the inferior oblique muscle (mm^2^)	48.02 (31.02–65.01)	27.17 (20.78–33.55)	< 0.001^*^
OM (mm^2^)	298.77 ± 83.31	169.18 ± 28.57	< 0.001^*^
CSA of the lacrimal gland (mm^2^)	91.20 ± 26.26	66.44 (53.36–79.51)	< 0.001^*^
IOFV(mm^3^)	6920.85 (5304.56–8537.13)	7640.08 (6790.41–8489.76)	0.183
Intraorbital fat density (HU)	-74.00 (-78.00–-70.00)	-85.00 (-89.00–-81.00)	< 0.001^*^
Vitreous body density (HU)	11.50 (9.50–13.50)	13.41 ± 2.92	< 0.001^*^
Lacrimal gland density (HU)	30.56 ± 12.21	33.50 (24.85–42.15)	0.368
IOFV/TV	0.30 (0.24–0.37)	0.33 (0.30–0.37)	0.018
OM/TOA	0.38 ± 0.08	0.27 ± 0.04	< 0.001^*^
CSA of the lacrimal gland/TOA	0.12 ± 0.04	0.11 (0.08–0.13)	0.107

*TAO*, thyroid-associated orbitopathy; *CSA*, cross-sectional area; *EOMs*, extraocular muscles; *IOFV*, intraorbital fat volume; *LGV*, lacrimal gland volume; *HU*, Hounsfield unit; *TV*, total orbital volume; *OM*, total cross-sectional area of extraocular muscles; *TOA*, total orbital cross-sectional area. ^*^p < 0.017 (the *p*-values were corrected using the Bonferroni method [corrected *α* = 0.05/3 ≈ 0.017; *p* < 0.017 was considered significant]). Data are presented as mean ± SD (normal distribution) or median (IQR) (non-normal distribution).

**Table 6:** Mann–Whitney *U* test between the inactive TAO group and the control group.

Parameters	Inactive TAO	Control	*P*-value
Exophthalmos (mm)	20.45 ± 3.10	15.46 ± 1.80	< 0.001^*^
CSA of the superior rectus muscle (mm^2^)	39.94 (31.14–48.75)	30.85 ± 7.45	< 0.001^*^
CSA of the inferior rectus muscle (mm^2^)	51.87 (32.74–70.99)	33.83 ± 8.17	< 0.001^*^
CSA of the internal rectus muscle (mm^2^)	35.63 (23.51–47.76)	27.75 (23.55–31.94)	< 0.001^*^
CSA of the superior oblique muscle (mm^2^)	15.71 (12.35–19.07)	12.10 (9.35–14.85)	< 0.001^*^
CSA of the inferior oblique muscle (mm^2^)	41.50 (30.70–52.30)	27.17 (20.78–33.55)	< 0.001^*^
OM (mm^2^)	237.23 (191.95–282.51)	169.18 ± 28.57	< 0.001^*^
CSA of the lacrimal gland (mm^2^)	81.67 (64.31–99.03)	66.44 (53.36–79.51)	< 0.001^*^
IOFV(mm^3^)	8,966.41 ± 2,638.90	7640.08 (6790.41–8489.76)	0.002^*^
Intraorbital fat density (HU)	− 85.61 ± 8.48	-85.00 (-89.00–-81.00)	0.866
Vitreous body density (HU)	11.00 (8.50–13.50)	13.41 ± 2.92	< 0.001^*^
Lacrimal gland density (HU)	28.00 (19.00–37.00)	33.50 (24.85–42.15)	0.005^*^
IOFV/TV	0.39 ± 0.10	0.33 (0.30–0.37)	< 0.001^*^
OM/TOA	0.27 (0.21–0.33)	0.27 ± 0.04	0.220
CSA of the lacrimal gland/TOA	0.10 (0.08–0.12)	0.11 (0.08–0.13)	0.225

*TAO*, thyroid-associated orbitopathy; *CSA*, cross-sectional area; *EOMs*, extraocular muscles; *IOFV*, intraorbital fat volume; *LGV*, lacrimal gland volume; *HU*, Hounsfield unit; *TV*, total orbital volume; *OM*, total cross-sectional area of extraocular muscles; *TOA*, total orbital cross-sectional area. ^*^p < 0.017 (the *p*-values were corrected using the Bonferroni method [corrected *α* = 0.05/3 ≈ 0.017; *p* < 0.017 was considered significant]). Data are presented as mean ± SD (normal distribution) or median (IQR) (non-normal distribution).

### Point-biserial correlation analysis

The results showed that the CSA of the superior rectus and medial rectus muscles, OM, orbital fat density, and the OM/TOA ratio in the three groups were positively correlated with the activity status of TAO (*r* > 0; *p* < 0.05) (see **Table 7** for details).

**Table 7:** Analysis of influencing factors associated with active TAO.

Parameters	Correlation coefficient (*r*)	*P-*value
CSA of the superior rectus muscle (mm^2^)	0.393	< 0.001^*^
CSA of the internal rectus muscle (mm^2^)	0.185	0.035^*^
OM (mm^2^)	0.316	< 0.001^*^
Intraorbital fat density (HU)	0.464	< 0.001^*^
OM/TOA	0.456	< 0.001^*^

A positive correlation (*r* > 0) indicates that the parameter value increases as the level of activity decreases, whereas a negative correlation (*r* < 0) signifies the opposite trend. *TAO*, thyroid-associated orbitopathy; *CSA*, cross-sectional area; *OM*, total cross-sectional area of extraocular muscles; *TOA*, total orbital cross-sectional area; *HU*, Hounsfield unit. ^*^p < 0.05.

### Diagnostic efficacy of orbital soft tissue parameters for TAO

**Figure 2:**
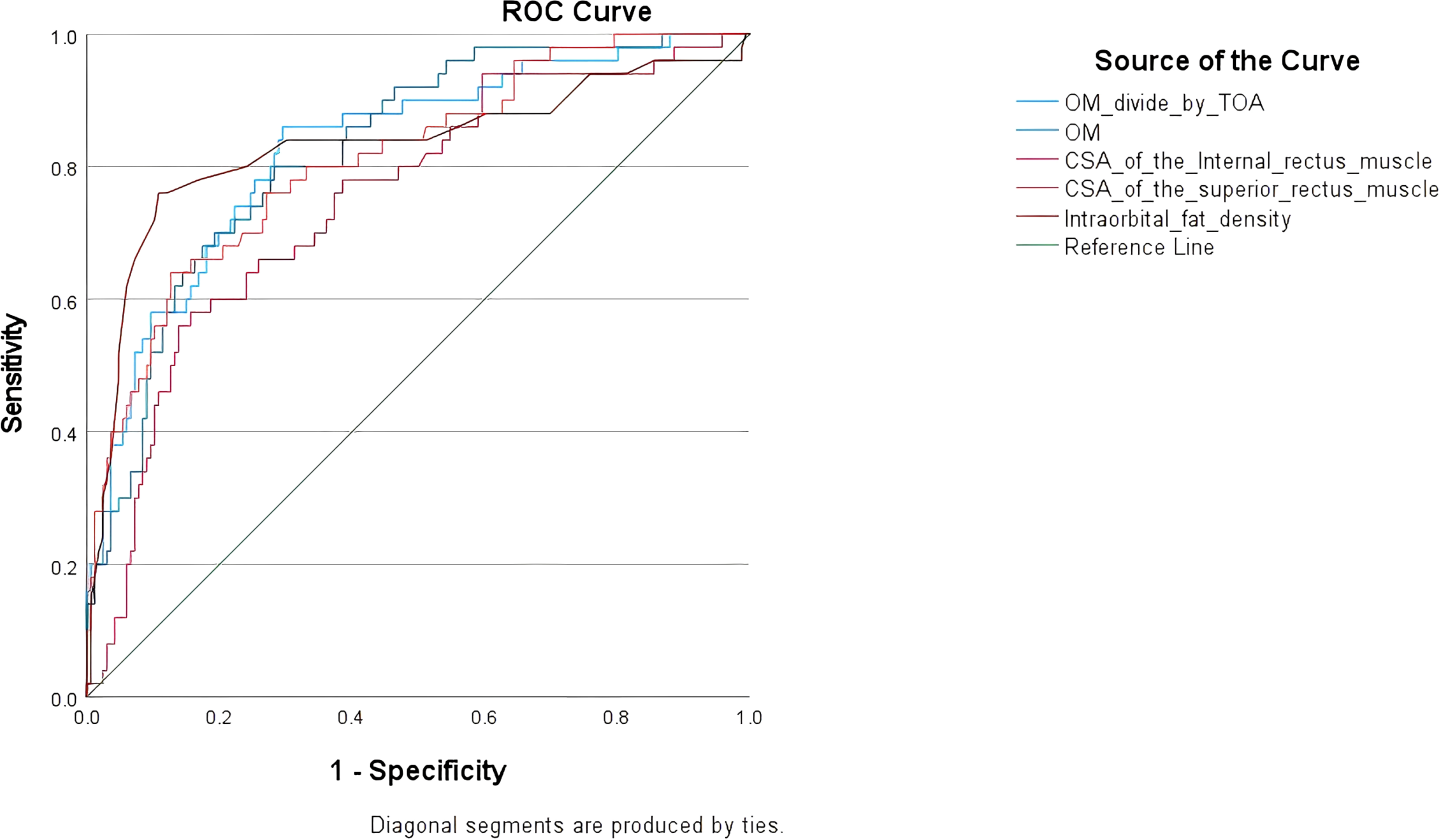
ROC curve. The AUC of intraorbital fat is the largest, as evident from the image. ROC, receiver operating characteristic; CSA, cross-sectional area; OM, total CSA of extraocular muscles*;* TOA, total orbital CSA; AUC, area under the curve.

All evaluated orbital indicators demonstrated statistically significant diagnostic value for TAO (all P < 0.001; **Table 8**), with area under the curve (AUC) values ranging from 0.749 to 0.830 (**Figure 2**, indicating good diagnostic efficacy). Intraorbital fat density yielded the highest AUC (0.830, 95% confidence interval [CI]: 0.751–0.909), followed by OM/TOA (AUC = 0.829, 95% CI: 0.762–0.895), OM (AUC = 0.825, 95% CI: 0.762–0.887), CSA of the superior rectus muscle (AUC = 0.812, 95% CI: 0.742–0.881), and CSA of the internal rectus muscle (AUC = 0.749, 95% CI: 0.671–0.826).

**Table 8:** ROC curve analysis results.

Test result variable(s)	AUC	Significant	95% CI	
			Lower	Upper
OM/TOA	0.829	0.000	0.762	0.895
OM	0.825	0.000	0.762	0.887
CSA of the internal rectus muscle	0.749	0.000	0.671	0.826
CSA of the superior rectus muscle	0.812	0.000	0.742	0.881
Intraorbital fat density	0.830	0.000	0.751	0.909

*ROC*, receiver operating characteristic curve; *AUC*, area under the curve; *OM*, total cross-sectional area of extraocular muscles; *TOA*, total orbital cross-sectional area; *CSA*, cross-sectional area.

Optimal diagnostic thresholds, sensitivity, specificity, and Youden indices for each indicator are summarized in **Table 9**. Intraorbital fat density had the highest Youden index (0.652) at a cut-off value of −78.5 (sensitivity: 76.0%, specificity: 89.2%). OM/TOA exhibited the highest sensitivity (86.0%) at a cut-off of 0.301 (specificity: 70.5%, Youden index: 0.565). For OM, the cut-off values were 43.52 for internal rectus muscle (sensitivity: 58.0%, specificity: 84.3%, Youden index: 0.423) and 49.11 for superior rectus muscle (sensitivity: 64.0%, specificity: 87.3%, Youden index: 0.513). OM had a cut-off value of 25,494.12 (sensitivity: 68.0%, specificity: 82.5%, Youden index: 0.505).

**Table 9:** ROC curve diagnostic threshold (cut-off value) analysis.

Test result variable(s)	Sensitivity	Specificity	Cut-off value	Youden index
OM/TOA	86.0%	70.5%	0.301	0.565
OM	68.0%	82.5%	25,494.12	0.505
CSA of the internal rectus muscle	58.0%	84.3%	43.525	0.423
CSA of the superior rectus muscle	64.0%	87.3%	49.115	0.513
Intraorbital fat density	76.0%	89.2%	− 78.5	0.652

*ROC*, receiver operating characteristic curve; *OM*, total cross-sectional area of extraocular muscles; *TOA*, total orbital cross-sectional area; *CSA*, cross-sectional area.

## Discussion

TAO is an ocular lesion mainly associated with autoimmune diseases, often presenting with local inflammatory ocular signs (11) and usually affecting both eyes simultaneously. The incidence rate is higher in women; however, the probability of men developing severe disease is four times that of women (12). In this study, the majority of TAO patients (92.5%) had bilateral involvement, but the gender distribution was relatively balanced (men:women = 35:32), which might be related to the relatively small sample size.

CT can clearly display the anatomical structure of the orbit, which is helpful for the diagnosis of TAO and has the advantages of being fast, convenient, economical, and highly repeatable. Currently, there are few studies on the use of CT values to evaluate the activity of TAO. No consensus has been reached on whether the quantitative analysis of orbital soft tissue parameters by CT can serve as a quantitative standard for evaluating the activity of TAO, and further research is needed. Previous studies mostly used the parameters of orbital soft tissue at 2mm behind the globe as the measurement basis (13). In clinical observation, this study found that 7 mm behind the globe is the position of the maximum CSA of the inferior rectus muscle, and the anatomical structure at this level is clear, making measurement easy to perform and the results highly accurate and repeatable. Therefore, the measurement values at 7mm behind the globe were selected as the reference indicators.

As previously noted, the primary orbital CT findings of TAO are exophthalmos and EOMs involvement. Most TAO patients present with unilateral or bilateral EOMs involvement, with the inferior rectus being the most commonly affected, followed by the medial, superior, and lateral rectus muscles (6, 14). EOMs thickening is one of the signs used to determine the diagnosis and severity of TAO. Most studies focus on the measurement of the density, volume, thickness, and diameter of EOMs and lacrimal glands (15–17). This study comprehensively analyzed the diagnostic value of the diameter, CSA, volume, and density of orbital soft tissues for inflammatory activity in TAO patients. We found that the inflammatory response in the active stage of TAO was significantly positively correlated with the CSA of the superior rectus muscle and medial rectus muscle, OM, orbital fat density, and the OM/TOA ratio (all *r* > 0; *p* < 0.05).

Lü et al. (19) observed the CT manifestations of 60 orbits in 30 normal individuals and 30 patients with TAO confirmed by clinical and laboratory examinations. They found that the characteristic CT manifestations of TAO patients mainly included hypertrophy of EOMs and exophthalmos, suggesting the diagnostic value of CT in TAO. Byun et al. (8) selected 80 TAO patients and 40 healthy controls and performed orbital CT scans and three-dimensional image analysis. They measured the volume and density of orbital fat, EOMs, and lacrimal glands. The results showed that the average total volume of extraocular muscles and lacrimal glands in the active group was significantly larger than that in the inactive group, and the prediction accuracy of the regression model based on the total volume of EOMs, lacrimal glands, and orbital fat, as well as the density of orbital fat and lacrimal glands, for TAO activity was 84.5%. This study also found that the CSA of the superior rectus muscle, inferior rectus muscle, superior oblique muscle, inferior oblique muscle, OM, and the CSA of lacrimal glands in TAO patients, were significantly higher than those in the normal control group, indicating that the CT imaging features of TAO patients were characterized by increased OM and lacrimal glands, which is consistent with the above research results. Differing from their observations, our study found that TAO patients had significantly lower vitreous density than normal controls. Reduced vitreous density is generally attributable to age-related, high myopia-related, or uveitis-induced alterations in vitreous structure and biochemical homeostasis. However, the age distribution was well balanced across groups in the present study; no case with high myopia; and no patients with concurrent uveitis were identified. Consequently, the primary cause underlying decreased vitreous density in patients with TAO could not be definitively determined. To date, no evidence has confirmed a causal relationship between TAO and reduced vitreous density, and further studies are warranted to validate this observation.

The increase in orbital fat is a significant clinical symptom of TAO (20). This study found that the orbital fat volume of TAO patients in the inactive stage was significantly larger than that of the normal control group, while there was no statistically significant difference between TAO patients in the active stage and the control group. The clinical course of TAO can be divided into two stages: the active stage (characterized by mononuclear cell infiltration of ocular tissues, accompanied by proliferation and edema of fibroblasts in EOMs, lacrimal glands, and fat tissues) and the inactive stage (mainly characterized by muscle fibrosis, and patients usually gradually enter this stage after 18 to 24 months of disease activity) (21). This is largely consistent with the findings of the present study: patients with inactive TAO typically exhibit a longer disease duration, whereas those with active TAO have a shorter disease course, which aligns with the natural progression of TAO from the active to the inactive phase. At the cellular and molecular levels, the differences in fat and muscle involvement may be related to differences in fibroblast phenotypes and cytokine profiles in different regions, the different T-cell subsets in the orbit during the disease course, the polymorphism of peroxisome proliferator-activated receptor-γ, and regulation of 11β-hydroxysteroid dehydrogenase-1. Muscle enlargement is clearly an early phenomenon in GO, while the increase in fat volume occurs relatively late (22). The above results may suggest that, during the active stage of the disease, orbital fat volume has not changed significantly; however, after entering the inactive stage, the volume of fat tissue may increase significantly due to metabolic or structural remodeling following chronic inflammation. This study suggests that orbital fat volume may be used to distinguish TAO patients in the active stage from those in the inactive stage. Specifically, an orbital fat density exceeding − 78.5 HU should raise high suspicion of active disease.

Increased intraorbital pressure (IOP) is one of the important characteristics of GO (23). This study found that exophthalmos in TAO patients was significantly higher than that in the control group, suggesting that it may be related to elevated intraorbital pressure. Some scholars have reported that there are significant differences in orbital fat structure between TAO patients and healthy controls, manifested as self-compression of fat cells, leading to increased orbital fat density in TAO patients (24). They also found that the increase in orbital fat density was correlated with a decrease in fat volume and an increase in EOMs volume. Researchers further speculated that the expansion of EOMs volume led to a reduction in available space within the orbit, thereby causing compression of fat tissue, while the orbital connective tissue bundles remained structurally intact during this process. Due to the strong X-ray absorption capacity of these connective tissue bundles, this may explain the phenomenon of increased density in the “fat cavities” observed in imaging. The results of this study showed that the orbital fat density of TAO patients in the active stage was significantly higher than that of patients in the inactive stage and the control group, while there was no statistically significant difference in fat density between patients in the inactive stage and the control group. In contrast, the orbital fat volume of TAO patients in the inactive stage was significantly larger than that of patients in the active stage and the normal control group. Additionally, OM in TAO patients in the active stage was significantly larger than that in patients in the inactive stage and the control group. When the OM value exceeds 25,494.12 mm^2^, TAO should be suspected as being in the active phase. In patients with active TAO, IOP is elevated, and enlargement of the extraocular muscles further compresses orbital adipose tissue, resulting in reduced adipose volume and increased adipose density. These morphological alterations help elucidate the underlying mechanisms of this pathological process and further validate the conclusions of previous related studies.

As noted above, in TAO, enlargement of the EOMs most commonly involves the inferior rectus, followed by the medial rectus, superior rectus, and lateral rectus (6, 14, 19). The results of this study show that inferior rectus muscle thickening is the most significant, followed by the superior rectus, inferior oblique, medial rectus, and lateral rectus muscles, which is broadly consistent with previous research findings. Unlike previous studies, we further identified that a CSA of the superior rectus muscle > 49.115 mm^2^ or a CSA of the medial rectus muscle > 43.525 mm^2^ should raise a high suspicion of active TAO. Le Moli et al. (25) calculated the OM and TOA of 23 normal controls and 32 GO patients through CT and found that CAS was positively correlated with the OM/TOA ratio, and improvement in CAS was strongly positively correlated with a decrease in the OM/TOA ratio, indicating that enlargement of the EOMs in the active stage of GO is an important clinical manifestation. This study found that the OM/TOA ratio of TAO patients in the active stage was significantly higher than that of the inactive stage group and the control group, and was significantly positively correlated with the CAS in TAO, further verifying the above research results. In addition, Tu et al. (18) retrospectively analyzed the orbital CT images of 116 TAO patients and 30 normal controls. The results showed that measuring the OM/TOA ratio at 2mm behind the globe on CT images can be used as an objective quantitative indicator for evaluating TAO activity; combined with CAS, it can help more accurately determine the disease state. This conclusion is consistent with the results of this study.

Tuo et al. (18), based on the area under the ROC curve analysis, concluded that when the OM/TOA ratio was ≥ 0.18, it had the best diagnostic efficacy for determining that TAO was in the active stage, with a sensitivity of 89.0% and a specificity of 53.5% (AUC = 0.761; *p* < 0.001). The results of this study showed that when the OM/TOA ratio was ≥ 0.301, the diagnostic efficacy was optimal, with a sensitivity of 86.0% and a specificity of 70.5% (AUC=0.829; *p* < 0.001). Although the sensitivity of the present study was slightly lower than that of Tuo et al.’s study (with a marginally increased risk of missed diagnosis), the specificity was significantly higher (with a lower rate of misdiagnosis). Additionally, the AUC of OM/TOA in our study was higher, indicating superior overall diagnostic efficacy.

This study has several limitations: First, the sample size of the included subjects is relatively small, and the study population is limited to a single academic medical center, without inclusion of a multicenter population, which may affect the representativeness and external validity of the research results. Second, as this study adopts a retrospective design, there may be selection bias and confounding factors, thereby imposing certain limitations on the interpretation and generalization of the results. Third, serial CT scans are neither safe nor practical, which substantially limits the utility of CT for longitudinal monitoring of disease activity. Lastly, CT is less sensitive and precise than MRI for evaluating soft tissue inflammation. The wide range and overlap of CT density values between active and inactive disease states further reduce the utility of CT measurements as a standalone diagnostic or monitoring tool. Fourth, there is an inherent limitation in using serial CT scans for assessing disease activity over time. Finally, the applicability of this study may be primarily restricted to primary healthcare institutions (i.e., facilities lacking advanced imaging equipment such as MRI).

## Conclusion

In summary, TAO patients are predominantly affected in both eyes and are more common in middle-aged and elderly individuals. The distinctive feature of the present study is that the measurement method employed is simple and straightforward, making it easily implementable in primary hospitals. Meanwhile, it demonstrates favorable sensitivity and specificity, thereby minimizing the risk of missed diagnosis or misdiagnosis in patients with active TAO. In the active stage group, the CSA of the superior rectus muscle and medial rectus muscle, OM, orbital fat density, and the OM/TOA ratio were all significantly higher than those in the inactive stage group and the control group, and these indicators were positively correlated with the disease activity state of TAO. In addition, these parameters can be used as reliable indicators for diagnosing active inflammation in TAO. To our knowledge, this study is one of the few that quantitatively analyzed the parameters of orbital soft tissues using plain CT to assess the feasibility of evaluating active inflammation in TAO. This method has important clinical reference value for diagnosing the active state of TAO in primary medical institutions.

## Data Availability

The original contributions presented in the study are included in the article/supplementary material. Further inquiries can be directed to the corresponding author.
